# Combining classifiers for robust PICO element detection

**DOI:** 10.1186/1472-6947-10-29

**Published:** 2010-05-15

**Authors:** Florian Boudin, Jian-Yun Nie, Joan C Bartlett, Roland Grad, Pierre Pluye, Martin Dawes

**Affiliations:** 1 DIRO, University of Montreal, CP. 6128, succursale Centre-ville, Montreal, H3C 3J7 Quebec, Canada; 2 Department of Family Medicine, McGill University, 515 Pine Avenue, Montreal, H2W 1S4 Quebec, Canada

## Abstract

**Background:**

Formulating a clinical information need in terms of the four atomic parts which are Population/Problem, Intervention, Comparison and Outcome (known as PICO elements) facilitates searching for a precise answer within a large medical citation database. However, using PICO defined items in the information retrieval process requires a search engine to be able to detect and index PICO elements in the collection in order for the system to retrieve relevant documents.

**Methods:**

In this study, we tested multiple supervised classification algorithms and their combinations for detecting PICO elements within medical abstracts. Using the structural descriptors that are embedded in some medical abstracts, we have automatically gathered large training/testing data sets for each PICO element.

**Results:**

Combining multiple classifiers using a weighted linear combination of their prediction scores achieves promising results with an *f*-measure score of 86.3% for P, 67% for I and 56.6% for O.

**Conclusions:**

Our experiments on the identification of PICO elements showed that the task is very challenging. Nevertheless, the performance achieved by our identification method is competitive with previously published results and shows that this task can be achieved with a high accuracy for the P element but lower ones for I and O elements.

## Background

Helping physicians to formulate their clinical information needs thorough well-built, focused questions and is one critical process of evidence-based practice (EBP) [1,2]. Without a well-focused question, it is more difficult and time consuming to identify appropriate resources and search for an answer [1]. Classical EBP teaching suggests that clinical questions can be separated in terms of four anatomic parts: Population/Problem (P), Intervention (I), Comparison (C) and Outcome (O), known as PICO elements [2]. For example, the question "*In children with an acute febrile illness, what is the efficacy of therapy with acetaminophen or ibuprofen in reducing fever?" *can be formulated as:

• Population/Problem: *children/acute febrile illness*

• Intervention: *acetaminophen*

• Comparison: *ibuprofen*

• Outcome: *fever*

Formulating a well-focused question according to the PICO framework facilitates searching for a precise answer within a large medical database [1]. However, using PICO terms in the information retrieval process is not straightforward. It requires the search engine to have detected and indexed PICO elements in the collection in order for the system to retrieve relevant documents. To our knowledge, no system has undertaken this level of indexing. In our pilot work we demonstrated that PICO elements are found in nearly all abstracts [3].

In terms of detecting PICO elements, it is not practical to annotate these elements at the phrase level due to significant un-resolvable disagreement and inter-annotator reliability issues [4]. This is why most previous work has focused on identifying PICO elements at a sentence level.

To date there is no satisfactory method of accurately predicting PICO elements from a corpus. In this study, we tested multiple supervised classification algorithms and their combinations for detecting PICO statements within medical abstracts. In continuation of previous work, we proposed to tackle the issue of extracting PICO elements in medical abstracts as a classification task and investigated the challenges of detecting these elements at the sentence-level.

Several previous approaches have reported promising results when categorizing sentence types in medical abstracts using classification tools [5-8]. Knight *et al. *[5] showed that Machine Learning could be applied to label structural information of sentences (i.e. Introduction, Method, Results or Conclusion) using a combination of relative sentence position and word distribution.

Demner-Fushman and Lin [6] have presented a method that use either manually crafted pattern-matching rules or a combination of basic classifiers to detect PICO elements in medical abstracts. Prior to that, the Metamap [9] program is used to annotate biomedical concepts in abstracts while relations between these concepts are extracted with SemRep [10], both tools being based on understanding the Unified Medical Language System (UMLS). The method described by Demner-Fushman and Lin obtained interesting results with an accuracy of 80% for predicting when the phrase contains a description of the *population *and *intervention*, 86% for *problem *and between 68% and 95% for *outcome*. It has to be noted that these scores are difficult to put into context due to the modest size of the test corpus (143 abstracts for *outcome *and 100 abstracts for other elements). Most of the errors are related to complementary processing such as inaccurate sentence boundary identification, chunking, Part-Of-Speech (POS) tagging or word sense disambiguation in the Meta-thesaurus. Based on this observation, we decided not to rely on software leveraging semantic knowledge resources.

Recently, supervised classification was proposed by Hansen *et al. *[7] to extract the number of trial participants. Results reported in their study show that the linear Support Vector Machine (SVM) algorithm achieves the best results with an *f*-measure of 86%. This may not be representative of a real-world task with only 75 highly topic-related abstracts used as testing set. Chung [8] extended this work to I and O elements using Conditional Random Fields (CRF). To overcome data sparseness, PICO-structured abstracts were automatically gathered from Medline in order to train and test classifiers. Experiments on a manually annotated test set (318 abstracts) show that promising results were obtained (*f*-measure of 83% for I and 84% for O) [11].

However, this study has several weaknesses. First, performance for each PICO element is computed in conjunction with the four generic rhetorical role classes (i.e. Aim, Method, Results and Conclusion). This methodology introduces bias by removing sentence candidates (sentences containing PICO elements are considered to occur only in the method section). Moreover, the rhetorical roles of previous, current and next sentences are included in the features used for classification, while in many medical documents these roles are not explicitly indicated, thus unavailable. Second, as POS tags are used as features, errors committed by the tagger will result in erroneous feature extraction. Finally, by using words as features and by including previous/following sentences feature sets, sentences are characterized by very high-dimensional feature vectors, which require high computational costs for their processing.

## Methods

PICO elements are more often implicitly described in medical documents. One can use linguistic patterns for this. However, the rule/pattern-based approach may require a large amount of manual work, and the robustness has yet to be proved on a large dataset. In this study, we tested a robust statistical classification approach, which requires less manual preparation.

### Construction of training and test data

Using supervised machine learning techniques requires both training and testing data sets. This is one major issue as the task of collecting data in a specialized domain has to be supervised by domain experts. This is also the reason why previous studies have been based on a small set of abstracts in tests. One solution is to use the structural information embedded in some abstracts for which the authors have clearly stated distinctive sentence headings. Some abstracts do contain explicit headings such as "PATIENTS", "SAMPLE" or "OUTCOMES", that can be used to locate sentences corresponding to PICO elements. Below is a segment of a document extracted from Medline using the PubMed http://www.ncbi.nlm.nih.gov/pubmed interface (PMID: 19318702) that includes PICO elements that are clearly identified:

*[...] ****PARTICIPANTS****: 2426 nulliparous, non-diabetic women at term, with a singleton cephalic presenting fetus and in labour with a cervical dilatation of less than 6 cm*. ***INTERVENTION****: Consumption of a light diet or water during labour*. ***MAIN OUTCOME MEASURES****: The primary outcome measure was spontaneous vaginal delivery rate. Other outcomes measured included duration of labour [...]*

The sentences under the headings can be easily extracted and used as the gold standard. We have extracted 260,000 abstracts from PubMed by stating the following search limits: *publication date 1999-2009, Humans, Clinical Trial, Randomized Controlled Trial, English *(Search conducted 2009/03/27). Four lists of distinctive headings, one for each PICO element, were manually created. For example, P headings contain POPULATION, PARTICIPANTS, PATIENTS, SUBJECTS, SAMPLE, etc. Afterwards, abstracts containing distinctive sentence headings were automatically selected and the first sentences following the PICO descriptor marked with corresponding PICO elements. Separating the Intervention and Comparison elements is an ambiguous task as they are referring to a same semantic group (exposures). For example, in a study that compares two drugs, it is often difficult to identify which drug is the intervention and which is the comparison. The abstracts corresponding to I and C are then regrouped in one data set. From the abstracts that were extracted, three data sets have been constructed (Table 1). Note that the abstracts could also contain sentences under other non PICO headings (e.g. "METHODS", "CONCLUSION"), which we did not include in our extraction process. Therefore, it was possible that no Outcome is extracted from a document by our process. This conservative extraction approach allowed us to obtain a dataset with as little noise as possible. Our testing data set contains 14279 abstracts for P, 9095 abstracts for I and 2394 abstracts for O against 318 abstracts for P, I and O in [8], and 90 for P, I and 143 for O in [6].

**Table 1:** Statistics about the training data

Dataset	Abstracts	Sentences
Population/Problem	14,279	191,608

Intervention/Comparison	9,095	125,399

Outcome	2,394	32,908

### Features used for classification

Prior to classification, each sentence underwent pre-processing treatments that replaced words into their canonical forms. Alphanumeric numbers were converted to numeric numbers while each word appearance in a series of manually crafted cue-words/verbs lists was investigated. The cue-words and cue-verbs were determined manually, some examples are shown below:

• Cue-verbs: *conduct *(P), *recruit *(P), *randomize *(I), *prescribe *(I), *assess *(O), *record *(O)

• Cue-words: *population *(P), *group *(P), *placebo *(I), *treatment *(I), *mortality *(O), *outcome *(O)

In addition, three semantic type lists, generated from the MeSH http://www.nlm.nih.gov/mesh ontology, were used to label terms in sentences. These lists are composed with entry terms corresponding to a selection of subgroups belonging to semantic types "*Living Beings*", "*Disorders*" and "*Chemicals & Drugs*". Table 2 shows the UMLS semantic identifiers used to classify sentences. Both statistical and knowledge-based features were extracted (Table 3). The reason for using naive statistical features such as the number of punctuation marks is motivated by the fact that authors normally conceive their abstracts according to accepted rules that govern writing styles for quantitative research in most medical journals.

**Table 2:** Statistics about the semantic type lists.

	ULMS Semantic type identifiers	Terms
List 1 (Living Beings)	Age Group (T100), Family Group (T099), Group (T096), Human (T016), Patient or Disabled Group (T101), Population Group (T098)	716

List 2 (Disorders)	Acquired Abnormality (T020), Anatomical Abnormality (T190), Cell or Molecular Dysfunction (T049), Congenital Abnormality (T019), Disease or Syndrome (T047), Experimental Model of Disease (T050), Finding (T033), Injury or Poisoning (T037), Mental or Behavioral Dysfunction (T048), Neoplastic Process (T191), Pathologic Function (T046), Sign or Symptom (T184)	23,541

List 3 (Chemicals & Drugs)	Amino Acid, Peptide, or Protein (T116), Antibiotic (T195), Biologically Active Substance (T123), Biomedical or Dental Material (T122), Carbohydrate (T118), Chemical (T103), Chemical Viewed Functionally (T120), Chemical Viewed Structurally (T104), Clinical Drug (T200), Eicosanoid (T111), Element, Ion, or Isotope (T196), Enzyme (T126), Hazardous or Poisonous Substance (T131), Hormone (T125), Immunologic Factor (T129), Indicator, Reagent, or Diagnostic Aid (T130), Inorganic Chemical (T197), Lipid (T119), Neuroreactive Substance or Biogenic Amine (T124), Nucleic Acid, Nucleoside, or Nucleotide (T114), Organic Chemical (T109), Organophosphorus Compound (T115), Pharmacologic Substance (T121), Receptor (T192), Steroid (T110), Vitamin (T127)	57,793

**Table 3:** Statistical features (marked with *) and knowledge-based (marked with †) features extracted for classifying sentences.

Feature
Position in the document (absolute, relative) *

Sentence length *

Number of punctuation marks *

Number of numeric numbers n > 10, n < 10 *

Word overlap with title *

Number of cue-words (P, I, O)†

Number of cue-verbs (P, I, O)†

MeSH semantic types

Number of (n = [0-9]+) †

### PICO Identification process

Tagging each document was performed in a three-step process. First, the document was segmented into plain sentences. Then each sentence was converted into a feature vector using the previously described feature set. Finally, each vector was submitted to multiple classifiers, one for each element, allowing the system to label the corresponding sentence. We used several algorithms implemented in the Weka toolkit http://www.cs.waikato.ac.nz/ml/: J48 and Random forest (decision trees), SVM (radial kernel of degree 3), multi-layer perceptron (MLP) and Naive Bayes (NB). For comparison, a position classifier (BL) was included as baseline in our experiments. This baseline method was motivated by the observation that PICO statements are typically found in specific sections of the abstract, which are usually ordered in Population/Problem, Intervention/Comparison and Outcome. Therefore, the relative position of a sentence could also reasonably predict the PICO element to which it is related. Similar methods to define baseline have been used in previous studies [5]. Demner Fushman et al [6], used the three first or last sentences of each abstract as the baseline. However, comparing classifiers that are restricted to label only one sentence per abstract with a multi-sentence baseline may lead to bias.

### Classification analysis

For each experiment, we report the precision, recall and *f*-measure of each PICO classifier. To paint a more realistic picture, 10-fold cross-validation is used for each classification algorithm. One round of cross-validation involves partitioning a sample of data into complementary subsets, performing the analysis on one subset (training set, 90% of the data), and validating the analysis on the other subset (testing set, 10% of the data). To reduce variability, 10 rounds of cross-validation were performed using different partitions, and the evaluation results were averaged over the rounds. Moreover, all sentence headings were removed from data sets converting all abstracts into unstructured ones. This treatment allowed us to have a more real-world scenario by avoiding biased values for features relying on cue-words lists.

The output of our classifiers is judged to be correct if the predicted sentence corresponds to the labelled one.

## Results

Performance of the five classification algorithms on each data set is shown in Table 4. No one classifier always outperforms the others but the multi-layer perceptron (MLP) achieves the best *f*-measure scores and SVM the best precision scores. We have performed more experiments on SVM with different kernels and settings. Best scores were obtained with a radial kernel of degree 3, other kernels giving lower scores or similar performance with higher computational costs.

**Table 4:** Performance of each classifier in terms of precision (p), recall (r) and f-measure (f).

	P-element			I-element			O-element		
	**p**	**r**	**f**	**p**	**r**	**f**	**p**	**r**	**f**

BL	52.1	52.1	52.1	21.9	21.9	21.9	20.0	20.0	20.0

J48	79.7	75.8	77.7	57.3	54.6	55.9	49.7	42.0	45.5
NB	66.9	65.0	66.0	50.1	47.9	49.0	48.6	47.7	48.1
RF	86.7	81.3	83.9	67.2	60.2	63.5	55.7	46.2	50.6
SVM	94.6	61.2	74.3	79.6	26.1	39.3	75.4	10.9	19.0
**MLP**	**86.3**	**84.5**	**85.4**	**67.1**	**65.6**	**66.3**	**57.0**	**54.5**	**55.7**

F1	89.9	78.2	83.6	71.2	55.2	62.2	62.6	42.7	50.8
F2	86.2	85.0	85.6	66.5	64.8	65.6	57.2	54.8	56.0
**F3**	**86.9**	**85.7**	**86.3**	**67.8**	**66.3**	**67.0**	**57.7**	**55.7**	**56.6**

As different classification algorithms performed differently on different PICO elements, in the second series of experiments, we used three strategies to combine classifier's predictions. The first method (F_1_) used voting: sentences that have been labelled by the majority of classifiers were considered candidates. In case of ambiguity (i.e. multiple sentences with the same number of votes), the average of the prediction scores were used to make a decision. The second and third methods computed a linear combination of the predicted values in an equi-probable scheme (F_2_) and using weights empirically fixed according to the observed *f*-measure ranking (F_3_) (i.e. for the P element: 5 for MLP, 4 for RF, 3 for J48, 2 for SVM and 1 for NB).

Combining multiple classifiers using F_3 _achieved the best results with a *f*-measure score of 86.3% for P, 67% for I and 56.6% for O. This strategy always outperformed, in terms of *f*-measure, the best classifier alone.

Similarly to [6], we then experimented our method at two and three sentences cut-off. Using the classifier's outputs as a ranking method and selecting the *n*-best sentences as candidates, we computed the *f*-measure scores for each classification algorithm on the Outcome element. Results are presented in Table 5. Best scores increased from 56.6% to 73.2% (2-sentence cut-off) and 80.6% (3-sentence cut-off).

**Table 5:** Performance of the Outcome classifiers in terms of f-measure (f) at 2 and 3 sentence cut-off.

	2-sentence cut-off	3-sentence cut-off
J48	57.0	61.2
NB	65.2	74.5
RF	61.9	67.3
SVM	19.1	19.1
**MLP**	**71.6**	**78.7**

F1	58.2	60.8
F2	71.4	78.8
**F3**	**73.2**	**80.6**

## Discussion

We tested a combination of classifiers to tackle the issue of sentence-level PICO element detection. Best results were obtained with a weighted linear combination of the prediction scores. Interestingly, not all fusing strategies always outperformed the best classifier alone. This is may be due to the high variation of performance between the classifiers.

Comparing our results with those published in previous studies is not an easy thing to do as testing data sets are different and therefore not directly comparable. However, considering that we performed 10-fold cross validation testing and that the size of our test data is considerably larger, our results suggest that this methodology tends to give more reliable results.

The O or I elements are more difficult to identify than P elements. The reason is not exclusively due to the decreasing amount of training data available but mainly to the task complexity. Indeed, I elements are often misclassified because of the high number of possible candidates. For example not only do drugs have a generic or ingredient term but may have several trade names. Terms belonging to the semantic groups usually assigned as I (e.g. drug names) are scattered throughout the abstract. Another reason is the use of non PICO-specific vocabulary, i.e. terms occurring in multiple PICO elements. For example, although treatments are highly related to intervention, they can also occur in other elements.

In the case of O elements, because abstracts generally contain more than one outcome, the data set we used for training is not really suited for the task. The fact that we used only one sentence per element, while building our training data, is a strong limiting factor. Sentence headings such as "OUTCOMES" clearly refer to several elements that are likely to be contained in more than one sentence. Previous work has shown that human annotators typically mark two of three sentences in each abstract as outcomes. Based on these observations, Demner Fushman [6] has proposed to evaluate the performance of the outcome classifier at a cut-off of two and three sentences. In the third series of experiments, we followed this assumption. Only SVM performance remained constant at the different sentence cut-off. This is due to the fact that the classifier produces binary prediction values that do not permit labelling more than one sentence with a statistically significant difference over the others. Results confirm that the strategy consisting of a weighted combination of the prediction scores (F_3_) always performs better. Although this evaluation roughly captures the performance of our classifiers, it shows that at a sentence cut-off of three, we are able to capture most of the outcomes.

Our experiments on the identification of PICO elements confirm that the task is very challenging. Using the structural descriptors that are embedded in some abstracts has allowed us to collect large data sets that would have been too costly and time-consuming to produce manually. The single sentence approach per element was very restrictive but a pragmatic approach. Tagging all the sentences that are under a heading is not a good solution either as the structural boundaries of an abstract can be vague. The question is, can we tolerate some noise in the training data? In case of a positive answer, one can think that the amount of training data can act as a smoothing by minimizing the impact of the false positive samples. But let us consider two examples (*PMID: 18265550 and 18263693*):

### Example 1

*[...] ****PATIENTS: ****In total 686 limbs in 574 patients at various clinical ...*

The clinical manifestations were categorized according to the CEAP ...

The distribution of venous insufficiency including the sapheno-femoral ...

The main duplex-derived parameters assessed were the reflux time ...

The venous reflux was assumed to be present if the duration of reflux was ...

The data obtained by APG were on VV (mL), VFI (mL/s), EF (%) and RVF ...

**RESULTS**: *There was no significant difference in overall superficial venous [...]*

### Example 2

*[...] ****PATIENTS: ****96 children, median (interquartile range) age 4.8 year...*

None received growth hormone treatment.

***MAIN OUTCOME MEASURES****: Two types of scoliosis were identified [...]*

In the first abstract, it is clear that considering all the sentences below the PATIENTS descriptor as P statements brings too many wrongly labelled samples. For the second example, the middle sentence about growth hormone treatment belongs to the P element; useful secondary information is contained in this but potentially more important information is in the first sentence.

It has to be noted that the features we used were not relying on manually crafted patterns or Part-Of-Speech tagging. Errors introduced by pre-processing were therefore not propagated to higher levels.

## Conclusion

In this study, we tested a robust statistical approach to PICO element detection within medical abstracts. The performance achieved by our identification method was competitive with previously published results in the overall precision of recall. The goal of this study was to understand if sentence level PICO detection was possible from a restricted set of features using Machine Learning techniques. Results showed that this task could be achieved with a high accuracy for the P element but not for I and O elements. The main issue remains in the evaluation. Having a sufficient number of manually annotated abstracts is now our priority. To this purpose, we are developing a web annotation tool that allows healthcare professionals to manually annotate Medline abstracts.

## Competing interests

The authors declare that they have no competing interests.

## Authors' contributions

FB carried out computer methods, performed data collection and preparation, and drafted the manuscript. MD participated in the writing of the manuscript. Other authors participated in the design and running of the study, and approved the final manuscript.

## Pre-publication history

The pre-publication history for this paper can be accessed here:

http://www.biomedcentral.com/1472-6947/10/29/prepub
